# La micro-abrasion amélaire associée à l’éclaircissement externe: intérêt dans la prise en charge de la fluorose

**DOI:** 10.11604/pamj.2019.34.72.20401

**Published:** 2019-10-04

**Authors:** Laïla Azzahim, Sanaa Chala, Faïza Abdallaoui

**Affiliations:** 1 Département d’Odontologie Conservatrice, Faculté de Médecine Dentaire de Rabat, Université Mohammed V, Rabat, Maroc; 2 Hôpital Militaire d’Instruction Mohammed V, Rabat, Maroc; 3 Laboratoire de Biostatistique, Recherche Clinique et Epidémiologie, Rabat, Maroc

**Keywords:** Micro-abrasion amélaire, éclaircissement externe, fluorose dentaire, Enamel microabrasion, external bleaching, dental fluorosis

## Abstract

La fluorose dentaire est une anomalie de développement qui affecte l'aspect esthétique des dents. L'association de la micro-abrasion à l'éclaircissement externe a montré des résultats satisfaisants pour l'amélioration de l'esthétique des dents atteintes de fluorose légère. L'objectif de ce travail est de mettre le point sur l'intérêt de cette association ainsi que sur ses différents effets sur la surface amélaire.

## Abstract (en)

Dental fluorosis is a developmental anomaly affecting aesthetic appearance. The association between microabrasion and external bleaching has shown satisfactory outcomes because it improves aesthetic outcome in patients with light dental fluorosis. The purpose of this study was to update the role of this association as well as its different effects on the enamelled surface.

## Introduction

La fluorose dentaire est une anomalie de développement qui affecte l'aspect esthétique des dents, causée par une consommation excessive et chronique de fluorure pendant l'odontogenèse. Les dents atteintes de fluorose présentent des lésions sous formes de tâches de couleurs variables (blanc crayeux, marron, brun) associées ou non à des pertes de substances [1]. Ce préjudice esthétique a un impact important sur la qualité de vie des patients concernés notamment en ce qui concerne leur intégration socioculturelle, ce qui impose une prise en charge adaptée [2]. L'objectif de ce travail est de mettre le point sur l'intérêt de la combinaison de la micro-abrasion à l'éclaircissement externe dans l'amélioration de l'esthétique des dents atteintes de fluorose légère ainsi que sur les différents effets de cette association sur la surface amélaire.

## Méthodes

Pour réaliser cette revue de littérature, nous avons effectué une recherche sur la base de données informatique PubMed (MEDLINE), en utilisant les mots-clés suivants: « enamel micro-abrasion », « dental micro-abrasion », « external bleaching », « dental bleaching », « fluorosis », « treatment », « association ». Ces mots-clés ont été combinés en autant d'étapes que nécessaire à l'aide de l'opérateur booléen « ET » (« AND »). Seuls les articles rédigés en langues française et anglaise ont été pris en compte. La lecture des titres et des résumés a permis de sélectionner les articles dans un premier temps, puis la lecture totale des articles sélectionnés a permis d'identifier les articles répondant à l'objectif de ce travail et ce quel que soit le type de l'étude.

## Etat actuel des connaissances

Pour traiter les dyschromies dues à la fluorose, plusieurs thérapies ont été proposées: la micro-abrasion, la macro-abrasion, l'éclaircissement externe, les restaurations prothétiques (facettes, couronnes…). Cependant, la plupart des patients sont jeunes et les options de traitement prothétique entraînent une élimination excessive de la structure de la dent à un âge précoce, en plus du coût élevé et des séances longues [2].

### Principe de la micro-abrasion

La micro-abrasion est un traitement chimio-mécanique qui consiste à appliquer un acide et un agent abrasif sur la surface de la dent affectée et qui est destinée à améliorer voire même éliminer les dyschromies limitées à la couche superficielle de l'émail [3]. L'épaisseur éliminée varie, selon les études, de 20 à 200 μm en fonction de la concentration en acide et de la durée de l'application [3]. Il est à noter aussi que les taches brunes sont généralement plus superficielles que les taches blanches. Ces dernières cèdent à la microabrasion amélaire dans environ 75% des cas en moyenne contre une réussite proche de 100% des cas pour les taches brunes [3]. Pendant la procédure de micro-abrasion, l'érosion acide et l'action abrasive des particules exercent un effet sur l'émail appelé l'effet d'« abrosion » attribuant à l'émail des caractéristiques histologiques et optiques particulières [4,5]. Sur le plan histologique, l'action érosive de l'acide aboutit à la désorganisation de la structure prismatique de l'émail. Lors de sa réorganisation, il y a production d'une matrice minérale en périphérie ce qui permet la formation d'une couche amélaire de surface correspondant à un émail aprismatique hautement compressé renforcée de particules issues du matériau de micro-abrasion (comme la silice) et/ou des pâtes de polissage (comme les fluorures) et qui se reminéralise progressivement au contact de la salive [6,7]. Sur le plan optique, l'effet d'« abrosion » permet d'obtenir une surface amélaire plus lisse sans irrégularités d'où l'aspect dit « glacé » ou « vernis ». En effet, la surface amélaire, étant formé d'émail aprismatique en périphérie, permet la réflexion et la réfraction de la lumière incidente, améliorant ainsi le rendu esthétique de l'émail, encore plus après hydratation de la dent par la salive. Ceci est expliqué par le fait que l'émail hypominéralisé est caractérisé par la présence de multiples interfaces séparant deux milieux d'indices de réfraction (IR) différents, respectivement IR=1,62 pour l'hydroxyapatite et IR=1,33 pour l'eau. Si la différence d'indice de réfraction est accentuée, la dispersion l'est aussi. C'est le cas lors du séchage des surfaces dentaires, chassant et remplaçant l'eau contenu dans une lésion par de l'air, d'indice de réfraction encore plus bas (IR proche de 1), l'hypominéralisation initialement non visible en milieu humide apparaît [5-7].

### Composition des matériaux utilisés pour la micro-abrasion

Plusieurs matériaux sont utilisés pour le traitement de la micro-abrasion. Prema^®^, Premier Dental Company (Philadelphia, PA, United States) contenant 10% d'acide chlorhydrique et des particules abrasives de carbure de silicium dont la granulométrie est de 30 à 60 μm et Opalustre^®^ (Ultradent, South Jordan, Utah, États-Unis), contenant 6,6% d'acide chlorhydrique et de microparticules de carbure de silicium d'une granulométrie de 20 à 160 μm, sont les produits les plus largement disponibles et les plus utilisés [4]. Le traitement de micro-abrasion s'effectue à l'aide de cupules spéciales en caoutchouc, montées sur contre-angle à basse vitesse de 300 tr/min, à raison de 10 secondes par dent et une force normalisée de 100 grammes, équivalente à 2 bars [8].

### Indications et limites de la micro-abrasion

Décrite à l'origine par Croll *et al.* (1989) [3], la micro-abrasion est réservée aux dyschromies limitées à la couche externe du tissu amélaire, sans implication de la dentine. Ainsi, la micro-abrasion amélaire peut être indiquée [9-11]: pour traiter la fluorose légère à modérée; pour corriger les irrégularités de la surface amélaire pouvant être secondaires à un traitement orthodontique, après retrait des matériaux de collage résiduels; pour traiter les différentes dyschromies de l'émail, blanches ou opaques, même avec des porosités, résultant du processus de déminéralisation/ reminéralisation. Cet aspect est commun aux lésions amélaires initiales retrouvées tout autour des brackets orthodontiques. Quoique ces tâches devront être traitées tout d'abord avec des agents de reminéralisation.

Les facteurs les plus importants contribuant au succès de la micro-abrasion de l'émail sont la localisation et la profondeur des dyschromies de l'émail [7, 10-12]. Cependant, il n'est pas évident de déterminer la profondeur des dyschromies car les moyens disponibles à l'heure actuelle sont très limités et fournissent peu de renseignements à ce sujet. Une source lumineuse de type LED placée au niveau de la face palatine ou linguale de la dent peut aider le clinicien à examiner l'émail. Ceci peut être utilisé pour estimer la profondeur de la lésion, car une couleur plus foncée indique une coloration plus profonde [7]. Un autre moyen rapporté par Park *et al.* (2016) [12] et qui ont proposé d'utiliser la Fluorescence Laser Quantitative (QLF) qui est un dispositif de diagnostic permettant de détecter les lésions carieuses précoces, car il peut analyser les lésions amélaires initiales (white spot) et donc aider à évaluer les limites de la micro-abrasion [12] mais ce moyen reste limité pour les autres cas de dyschromies. Les tâches opaques plus profondes, telles que celles résultant d'une hypoplasie ou des colorations d'origine génétique ou congénitale, ne peuvent pas être traitées par micro-abrasion et nécessitent une approche restauratrice d'où les limites de cette thérapeutique [11].

### Intérêt de l'association micro-abrasion/éclaircissement externe

**Figure 1:**
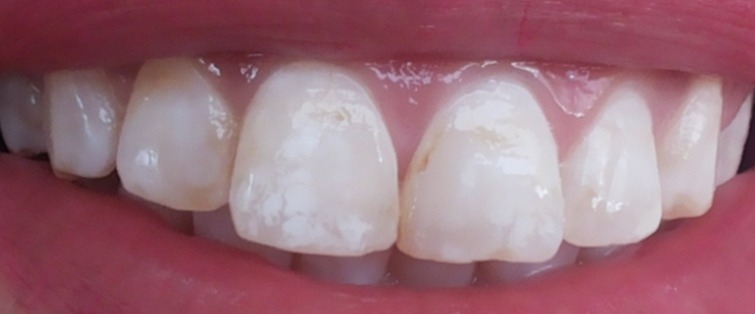
Photographie pré-opératoire montrant des taches blanches opaques et brunes au niveau des faces vestibulaires des dents antérieures

**Figure 2:**
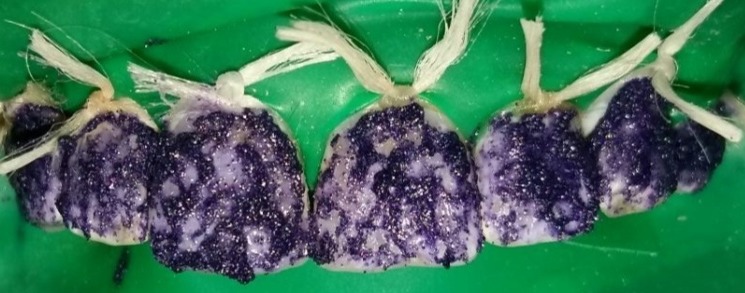
Application du matériau de micro-abrasion

**Figure 3:**
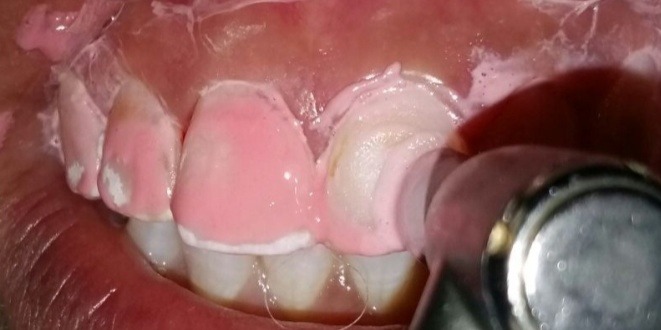
Réalisation d’un polissage à l’aide d’une pâte fluorée après rinçage

**Figure 4:**
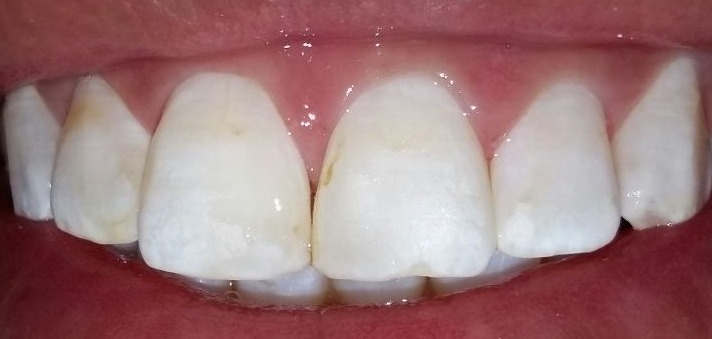
Résultat après la micro-abrasion

**Figure 5:**
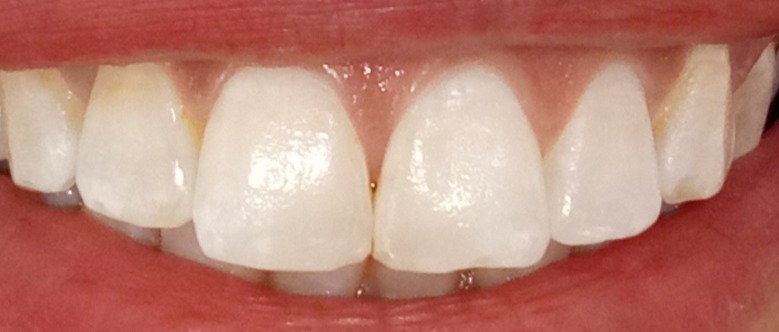
Résultat après éclaircissement externe

La perte d'épaisseur d'émail peut laisser transparaître la dentine sous-jacente, d'où l'aspect jaunâtre des dents après micro-abrasion. L'éclaircissement permettra alors de diminuer la saturation de la couleur [13,14]. Une autre indication de cette association est l'harmonisation de la couleur, puisque, dans les cas de fluorose, les dents présentent un aspect crayeux et nuageux qui peut être atténué par la micro-abrasion. L'éclaircissement externe permet d'optimiser le rendu esthétique en diminuant le contraste entre émail sain et émail tâché [13,14] (Figure 1, Figure 2, Figure 3, Figure 4, Figure 5). Plusieurs travaux ont étudié l'effet des différentes thérapeutiques non invasives de la fluorose légère sur l'émail dentaire, notamment l'effet de la micro-abrasion associée à l'éclaircissement externe. Les critères d'évaluation sont variables d'une étude à l'autre et les paramètres les plus étudiés sont: l'amélioration de l'aspect esthétique, la survenue d'effets secondaires, les altérations de la surface amélaire et la qualité du collage à l'émail.

### Effet de l'association micro-abrasion amélaire/éclaircissement externe en termes d'amélioration de l'aspect esthétique

C'est le paramètre le plus rapporté dans la littérature sans doute. La majorité des travaux publiés présente des résultats très satisfaisants montrant une amélioration nette de l'aspect esthétique après l'application de cette association [1,2,9-11,14]. Ce que l'on peut reprocher à ces travaux, c'est qu'il s'agit d'un faible niveau de preuve scientifique, puisqu'il s'agit souvent de rapport de cas et que les études à haut niveau de preuve scientifique (les essais contrôlés randomisés, les revues systématiques et les méta-analyses) restent peu nombreuses [15].

### Effet de l'association micro-abrasion/éclaircissement externe en termes de survenue d'effets secondaires: sensibilité dentaire et irritation gingivale

Castro *et al.* (2014) ont rapporté, dans un essai clinique randomisé [16], qu'il n'y avait pas de différence statistiquement significative entre les deux techniques (micro-abrasion seule et micro-abrasion associée à l'éclaircissement externe en ambulatoire), car dans les deux groupes de traitement, il y a eu une amélioration de l'aspect esthétique sans la survenue d'effets secondaires (les sensibilités dentaires ou l'irritation gingivale). Cependant, les patients ayant bénéficié d'un éclaircissement externe à domicile après un traitement micro-abrasif ont déclaré qu'ils étaient plus satisfaits de l'apparence de leurs dents [16]. Di Giovanni *et al.* (2018) ont réalisé une revue systématique [15], dans laquelle ils ont rapporté que la survenue d'effets indésirables (sensibilité dentaire et irritation gingivale) était transitoire et reste acceptable pour les interventions évaluées (micro-abrasion seule, éclaircissement externe en ambulatoire, association des deux et enfin l'infiltration résineuse).

### Effet de l'association micro-abrasion/éclaircissement externe sur la micro-dureté et la rugosité de l'émail

Il existe peu d'études évaluant l'effet de l'association entre la micro-abrasion et l'éclaircissement externe en ambulatoire sur la structure de l'émail. Dans une étude in vitro [17], Franco *et al.* (2016) ont rapporté que l'association de la micro-abrasion de l'émail à l'éclaircissement externe n'avait pas d'influence sur la micro-dureté ni sur la rugosité de surface des dents micro-abrasées, que ce soit avec une combinaison immédiate ou différée, car, selon la même équipe, l'éclaircissement externe des dents ayant subi la micro-abrasion n'accentuait pas les modifications morphologiques normalement observées sur une surface déjà traitée par micro-abrasion [17]. Cela peut s'expliquer par le fait que, dans le cadre de la technique de micro-abrasion, il est important de procéder à un polissage final afin d'optimiser l'aspect esthétique et de minimiser la rugosité de la surface de l'émail, car une rugosité supérieure entraîne une plus grande rétention de plaque. La technique utilisant les particules de silice donnerait une surface amélaire brillante et polie comme l'ont montré les observations sous microscope électronique à balayage dans l'étude de Pini *et al.* [7]. Ce dernier a également rapporté qu'il n'y a pas de différence statistiquement significative entre les acides utilisés (acide phosphorique à 35% et acide chlorhydrique à 6,6%) en termes de rugosité d'émail. Par ailleurs, l'état de surface amélaire est amélioré en présence de la salive, comme l'ont montré les études *in situ* et *in vitro* [6,7,17,18]. Ceci est dû à sa teneur en sels minéraux et son effet tampon grâce aux bicarbonates qu'elle contient. Pini *et al.* (2017) [18] expliquent que la silice (SiO_2_) présente dans le matériau de micro-abrasion, pourrait être incorporée dans l'émail après micro-abrasion. Ce composant est connu pour contenir un matériau bioactif, le silicate tricalcique (Ca_3_SiO_5_) qui induit la formation d'une nouvelle couche d'apatite sur l'émail préalablement déminéralisé par l'action de l'acide. Par conséquent, sa présence sur l'émail pourrait améliorer le processus de minéralisation, car elle pouvait se lier au calcium provenant de l'hydroxyapatite et de la salive, ce qui conduirait à la formation de nouveaux cristaux d'apatite capables de réduire et de résister au processus de déminéralisation [17,18]. A partir de ce principe (incorporation de silice ou de fluorures après la micro-abrasion), d'autres associations ont été proposées afin d'incorporer d'autres éléments permettant de reminéraliser l'émail et d'augmenter sa résistance à la déminéralisation.

### Autres associations

Di Giovanni *et al.* (2018) [15] rapportent que l'infiltration résineuse semble être plus efficace dans le traitement esthétique des dents atteintes de fluorose légère à modérée que l'éclaircissement externe et la micro-abrasion, seuls ou combinés. Gencer *et al.* (2019) [13] rapportent les mêmes constatations. De nombreux auteurs suggèrent la possibilité d'appliquer indifféremment un gel à base de fluorures ou un gel à base de « casein phosphopeptide-amorphous calcium phosphate (CPP-ACP) » en fin de microabrasion pour favoriser la reminéralisation et éviter les sensibilités postopératoires. Selon Deshpande *et al.* (2017) [8], l'association de micro-abrasion de l'émail à l'utilisation de CPP-ACP gel peut constituer un moyen très prometteur de traiter les taches blanches de l'émail (fluorose légère et hypominéralisation), les irrégularités, les défauts de développement et les lésions initiales résultant du traitement post-orthodontique.

## Conclusion

Dans le cas de la fluorose légère à modérée, l'association micro-abrasion/éclaircissement externe peut être un compromis raisonnable entre aspect esthétique acceptable, perte de substance minime, effets secondaires transitoires et coût abordable [19,20]. C'est le cas de la situation clinique illustrée dans ce travail, où le résultat de la micro-abrasion seule n'a pas répondu à la demande esthétique de la patiente tandis que l'association à l'éclaircissement externe a permis d'améliorer l'aspect esthétique et de satisfaire la patiente.

### Etat des connaissances actuelles sur le sujet

- L'association micro-abrasion amélaire/éclaircissement externe permet une amélioration satisfaisante de l'aspect esthétique des dents atteintes de fluorose légère.

### Contribution de notre étude à la connaissance

- Faible niveau de preuve scientifique des études disponibles sur les différents effets de l'association micro-abrasion amélaire et éclaircissement externe (il s'agit dans la plupart des articles de séries de cas ou d'études expérimentales);
- Nécessité de plus d'études avec un long recul clinique évaluant l'effet de cette association sur l'émail dentaire particulièrement sur le plan histologique.

## Conflits d’intérêts

Les auteurs ne déclarent aucun conflit d'intérêts.
